# Yttrium Oxide Freeze-Casts: Target Materials for Radioactive Ion Beams

**DOI:** 10.3390/ma14112864

**Published:** 2021-05-27

**Authors:** Eva Kröll, Miriana Vadalà, Juliana Schell, Simon Stegemann, Jochen Ballof, Sebastian Rothe, Doru C. Lupascu

**Affiliations:** 1 Institute for Materials Science and Center for Nanointegration Duisburg-Essen (CENIDE), University of Duisburg-Essen, 45141 Essen, Germany; miriana.vadala@uni-due.de (M.V.); juliana.schell@cern.ch (J.S.); doru.lupascu@uni-due.de (D.C.L.); 2 European Organization for Nuclear Research (CERN), CH-1211 Geneva, Switzerland; simon.thomas.stegemann@cern.ch (S.S.); jochen.ballof@cern.ch (J.B.); sebastian.rothe@cern.ch (S.R.)

**Keywords:** freeze casting, scaffolds, porous ceramic, yttrium oxide, target material, radioactive ion beams, ISOLDE

## Abstract

Highly porous yttrium oxide is fabricated as ion beam target material in order to produce radioactive ion beams via the Isotope Separation On Line (ISOL) method. Freeze casting allows the formation of an aligned pore structure in these target materials to improve the isotope release. Aqueous suspensions containing a solid loading of 10, 15, and 20 vol% were solidified with a unidirectional freeze-casting setup. The pore size and pore structure of the yttrium oxide freeze-casts are highly affected by the amount of solid loading. The porosity ranges from 72 to 84% and the crosslinking between the aligned channels increases with increasing solid loading. Thermal aging of the final target materials shows that an operation temperature of 1400 °C for 96 h has no significant effect on the microstructure. Thermo-mechanical calculation results, based on a FLUKA simulation, are compared to measured compressive strength and forecast the mechanical integrity of the target materials during operation. Even though they were developed for the particular purpose of the production of short-lived radioactive isotopes, the yttria freeze-cast scaffolds can serve multiple other purposes, such as catalyst support frameworks or high-temperature fume filters.

## 1. Introduction

**Figure 1:**
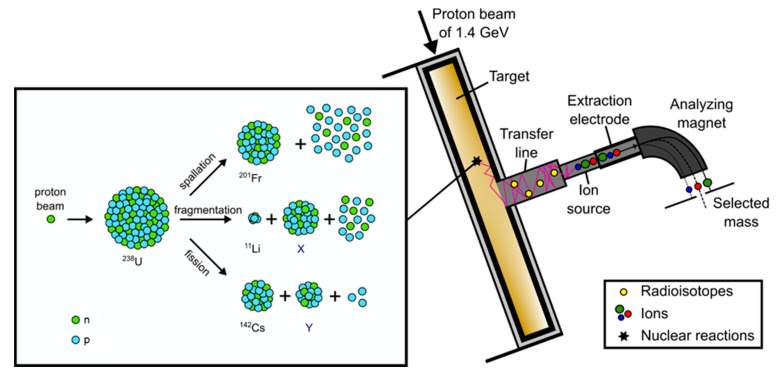
Schematic illustration of the ISOL method and nuclear reactions using ^238^U as an example.

ISOLDE [1] is a radioactive ion beam (RIB) facility, located at the Proton Synchrotron Booster (PSB) at CERN’s accelerator complex. Here, the Isotope Separation On Line (ISOL) method is applied to produce over 1000 radioactive isotopes of over 75 chemical elements via particle-induced nuclear reactions in thick target materials. The ISOLDE targets are placed in a resistively heated tantalum furnace connected to an ion source in a vacuum vessel (target and ion source unit) bombarded with a high-energy proton beam of 1.4 GeV, delivered from the PSB accelerator. By heating the target to high temperatures (maximum temperatures range up to 2000 °C) the released isotopes move through the target matrix to its surface, from which they are desorbed, followed by effusion to the ion sources, where they are charged to form typically singly charged positive ions. An extraction potential accelerates the ions to energies up to 60 keV, which allows them to be transferred via a dipole mass separator magnet (see Figure 1), selecting an isobar, and to the research beam lines distributed in the ISOLDE hall [2].

Depending on the isotope required by the particular ISOLDE experiment, the combination of target and ion source has to be deliberately selected. The choice depends on the physico-chemical properties of the element, defined by the number of protons in the nucleus (Z) and the half-life of the selected isobar given by the mass (A) of the isotope of interest. A second, but nonetheless important aspect is contaminants of other elements which could be detrimental to certain experiments. For the target material choice, several aspects have to be considered and weighed against another: nuclear reaction cross-section and density of the target versus high-temperature stability, chemical compatibility with the isotope of interest, and diffusion time. Further details can be found in the recent review by Ramos [3]. Typical target materials operated at ISOLDE are pure metal foils (25 mm thick tantalum, titanium), liquid metals (lead, tin), carbide pellets (uranium, silicon), or oxide fibers (thoria, zirconia).

Since the isotope release is often one of the main bottlenecks in exotic and/or intense RIB production [4], and since the isotope release is governed by diffusion and effusion processes [5,6], targets are usually operated at the highest temperature possible. The maximum temperature is mainly restricted by the vapor pressure of the target material and the sintering rate, which limits the operation time. Refractory ceramics are a group of materials which have advantageous properties in this respect [2,3] as well as the possibility to design the microstructure via methods used in this work. An open microstructure enables higher release and transport rates [3,4]. A conventional method to prepare solid targets is pressing, which forms randomly densified powders or fibers into pellets. In contrast with conventional uniaxial or isostatic pressing, freeze casting allows for the production of tailored microstructures with aligned pore channels [7]. This aligned microstructure determines the orientation of the release paths and increases the release rate. Czapski et al. successfully demonstrated that alumina and silicon carbide targets, which are prepared via the freeze-casting method, are suitable for the production of radioactive ion beams [8]. This work resumes our attempt to fabricate oriented pore structures in yttrium oxide freeze casts. Since the early 2000s, freeze casting has been a popular method to produce anisotropic and isotropic pore structures. These pore structures are obtained by a segregation process of solidifying carrier liquid and a second phase [9]. The final microstructure is a replica of the frozen liquid after its sublimation and is densified in a subsequent sintering process. Metals, polymers and ceramic materials, e.g., ZrO_2_, Al_2_O_3_, and TiO_2_ [10,11,12], act as second phase. Yttrium oxide, as a refractory material, has been used as target material at the ISOLDE facility to produce, for example, krypton beams [2,13]. There have been several studies focusing on the fabrication of transparent yttrium oxide [14,15] or the characterization of yttrium oxide slurries [16,17], but research on porous yttrium oxide structures [18] is rare, despite being a highly interesting material due to its high melting point (~2430 °C) and its thermal stability [19]. In the following, we will show the preparation of yttrium oxide freeze-casts as target materials and discuss the effect of solid loading and thermal aging on the scaffold structures. The deposited energy on the target exposed to the ISOLDE beam was simulated by FLUKA codes [20,21]. Thermal stresses were calculated using the simulated data and compared with the compressive strength of the prepared freeze casts, to estimate the mechanical integrity of the porous material during application.

## 2. Materials and Methods

Yttrium oxide targets were fabricated by the unidirectional freeze-casting method to obtain an anisotropic pore structure [7]. Aqueous suspensions were prepared with high purity Y_2_O_3_ powder (d_50_ = 5.04 µm, Alfa Aesar, Haverhill, MA, USA). Distilled water was used as carrier liquid and Dolapix CE64 (Zschimmer & Schwarz, Lahnstein, Germany) was added as an anionic dispersant. Polyvinyl alcohol (PVA, Sigma Aldrich, St. Louis, MO, USA) and polyethylene glycol (PEG, Fluka, Switzerland) acted as a binder to achieve sufficient green body strength. The solid loadings of the ceramic powder were 10, 15, and 20 vol% (volume with the respect to the total liquid volume). Table 1 shows the compositions of the prepared suspensions. For the sake of convenience, in subsequent paragraphs, the samples are referenced as listed in Table 1.

The suspensions were further ball-milled with zirconia balls for 72 h. The final suspensions were poured into polytetrafluoroethylene (PTFE) molds with an inner diameter of 12 mm and a height of 25 mm. The cylindrical molds were placed on a customized freeze casting device consisting of a brass cylinder cooled by liquid nitrogen. Due to the temperature gradient inside the molds, the suspension was frozen perpendicular (bottom-to-top) to the surface of the brass cylinder. The frozen samples were placed in a cold chamber at −21 °C for 24 h to secure the solidification. After freezing the suspension, the samples were lyophilized in a freeze-drying device at −20 °C and 0.3 mbar for 48 h. Subsequently, the yttria samples were heated up to 550 °C at a heating rate of 1 K min^−1^ and further held for 1 h to burn out the organic additives. Afterwards, the samples were heated up to the sintering temperature at a heating rate of 3 K min^−1^ and sintered at 1400 °C for 4 h in air.

To analyze the suspension properties, the resulting microstructure, and the porosity, various analyses were performed. The zeta potential of the suspension was measured using a zeta potential analyzer (Stabino I, Colloid Metrix, Meerbusch, Germany). To determine the viscosity, a rheometer (Rheostress RS75, Haake, Karlsruhe, Germany) was used. For microstructural characterization, the samples were cut and polished. The observation of the microstructure was made with a scanning electron microscope (SEM, Apero S, Thermo Fisher, Waltham, MA, USA). The average pore size was estimated via SEM images. Furthermore, the bulk density and open porosity were measured and calculated by Archimedes’ principle. Using mercury porosimetry (Pascal 440, Thermo Scientific, Waltham, MA, USA), the pore size distribution and pore structure of the samples was investigated. After aging the samples at 1400 °C for 4 days, SEM imaging was repeated. For the compressive strength measurements, samples with a diameter of 11 mm and a height of 15 mm were used. Three samples of each kind were loaded at 1 N s^−1^ (precision universal testing machine, AG-X plus, Shimadzu, Kyoto, Japan). FLUKA (version 4.1.0, CERN) was used to simulate the energy deposition on the prepared target materials. The measured compressive strength and the calculated thermal stress from the FLUKA data were compared to evaluate the mechanical suitability for the proposed application.

## 3. Results and Discussion

### 3.1. Suspension Properties and Sintering Shrinkage

**Figure 2:**
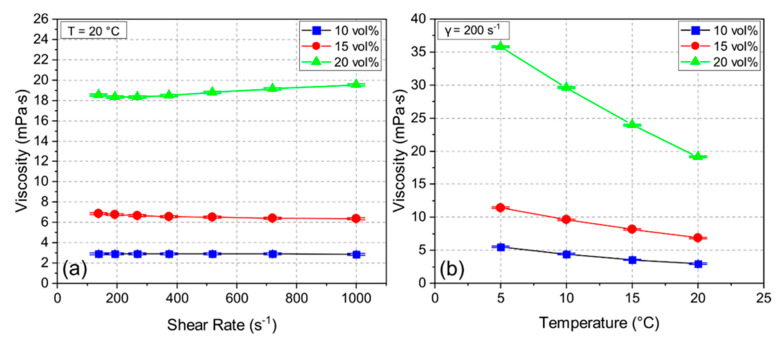
Viscosity of suspensions 10Y, 15Y, and 20Y at different shear rates (**a**) and different temperatures (**b**).

During the freeze casting process, the stability of the suspension is crucial to avoid early segregation. The zeta potential measurement of the ceramic suspension indicated negatively charged particles at a pH of 10.0 with a zeta potential of 27.3–38.2 mV, which yielded a high suspension stability during the whole process [22]. Figure 2 shows viscosity measurements for samples 10Y, 15Y, and 20Y with shear rates from 140 s^−1^ to 1000 s^−1^ at 20 °C (see Figure 2a) and the temperature-dependent viscosity measurements at a shear rate of 200 s^−1^ (see Figure 2b). For the samples 10Y and 15Y with low solid loading, the behavior was Newtonian. The 20Y sample showed slightly shear-thickening behavior. Figure 2a also indicates that the viscosity increased with increasing solid loading almost independent of the shear rate. Figure 2b shows the effect of temperature on the viscosity. For all three samples, the viscosity increased with decreasing temperature. This effect is more distinct for the 20Y suspension, where the viscosity increased from 19 mPa∙s to 36 mPa∙s, which can lead to an irregular growth of ice crystals during freeze casting [23].

After sintering, the shrinkage ranged from 5.9% to 6.6% and from 7.4% to 8.4% for the diameter and height of the samples, respectively (see Table 2). Due to the anisotropic pore structure, the shrinkage was similarly anisotropic. The green density of the initial samples affected the sintering shrinkage [24]. Hence, samples with higher solid loading and higher green density experienced less sintering shrinkage.

### 3.2. Effect of Solid Loading

**Figure 3:**
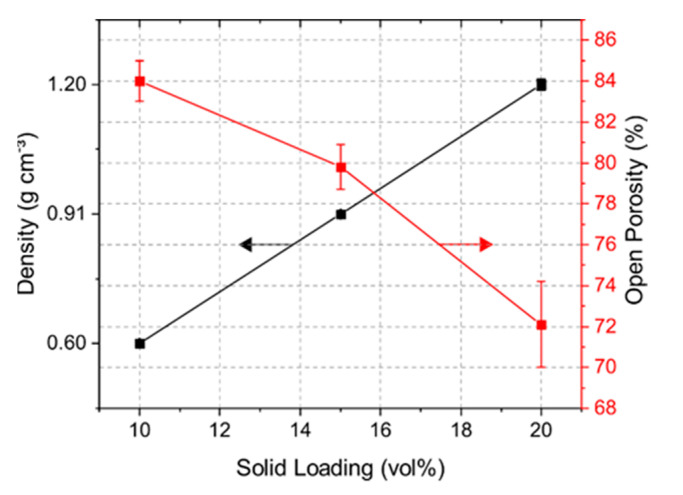
Density and open porosity determination via Archimedes’ principle.

Solid loading of a suspension had a large impact on the porosity and mechanical strength of the samples. Figure 3 shows the bulk density and open porosity of the samples with solid loading of 10, 15, and 20 vol% yttrium oxide. The 10Y samples had a density of 0.60 g cm^−3^, which rose with increasing solid content up to 1.20 g cm^−3^. The obtained open porosity for the 10Y, 15Y, and 20Y samples was 84, 80, and 72%, respectively. Closed porosity in the target material may lead to trapped isotopes in the pore volume, which reduces the isotope release. Comparing the measured results with the pure density of Y_2_O_3_ of 5.01 g cm^−3^, the closed porosity for the 10Y, 15Y and 20Y samples was less than 4%.

**Figure 4:**
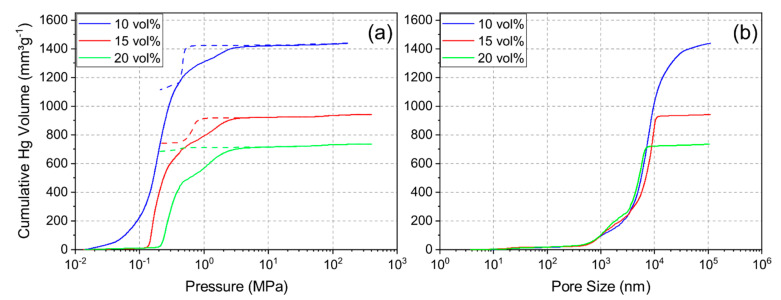
Intrusion and extrusion (dashed line) curves (**a**) and cumulative pore size distributions (intrusion) (**b**) for mercury porosimetry for samples 10Y, 15Y, and 20Y.

Mercury porosimetry was performed to determine the pore size distribution and examine the pore structure. Figure 4a shows the intrusion and extrusion curves of mercury and Figure 4b shows the resulting cumulative pore size distribution of each sample. The pore size distribution indicated a shift to smaller pore sizes and less porosity with increasing solid loading. The median pore diameter for the 10Y, 15Y and 20Y samples was 7.2 µm, 6.5 µm, and 4.1 µm, respectively. Additionally, the pore distribution became narrower with increasing solid loading, and the 20Y sample showed a slightly multimodal pore size distribution. Figure 4a shows typical examples for intrusion and extrusion hysteresis which open up with increasing solid loading. This phenomenon was seen for sample 15Y but was more distinct for sample 20Y. After complete depressurization, mercury was trapped in the internal pore network, which leads to an open hysteresis. Most likely explanations for this behavior are effects of the pore shape and pore connectivity [25,26]. Pore structures with a large ratio between the entrance and cavity size require more pressure for mercury extrusion [26]. Ink-bottle pores and pores with high connectivity are examples of such pore structures. This may be the reason for trapped mercury in the pore structure. Our data imply that the unidirectional pore structure of the freeze-casts contains cross-linked volumes, ink-bottle pores, or non-cylindrical pore channels. With an increase in solid loading, the crosslinking increases; hence, a larger fraction of the mercury is trapped in samples which were synthesized from slurries with increased solid loading.

**Figure 5:**
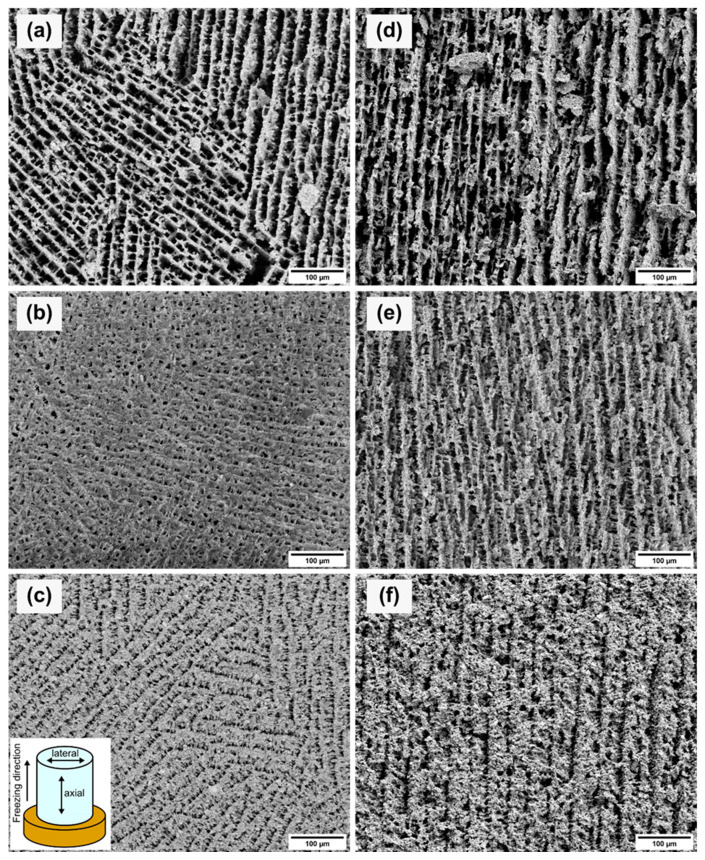
SEM images of the lateral (left) and axial (right) cross-sections of samples 10Y (**a**,**d**), 15Y (**b**,**e**), and 20Y (**c**,**f**). The axial cross-sections planes contain the direction of the unidirectional solidification.

Figure 5 shows SEM images of the axial and lateral cross-sections for each sample. Due to the gradient-controlled solidification of the samples, the lateral and axial cross-sections showed different projections on the pore structures. The lateral cross-sections (see Figure 5a–c) depict the pore network from the top view of the samples. The porosity and the pore size decreased with increasing solid loading. Therefore, the pore channels became narrower with increased solid loading. The measured pore sizes showed the same trend as the Archimedes measurements and mercury porosimetry. The average pore sizes are given in Table 3. The difference between the pore size values of the mercury porosimetry and the measurements of the SEM images is caused by the measuring principle of the mercury porosimetry. Mercury porosimetry results show smaller pore sizes than SEM results, because the former method determines the largest entrance to a pore instead of the internal pore size [26]. Figure 5a shows differently oriented areas consisting of parallel pore channels which are interrupted by thin walls. The pores of the 10Y sample displayed a rectangular shape. With increasing solid loading, the pores became square-shaped (see Figure 5b) and narrower (see Figure 5c). Figure 5c shows the same parallel ordered domains as Figure 5a. This multiple-domain structure is attributed to the ice crystals formed randomly during the solidification and the meeting interfaces of the ice fronts [27]. The axial cross-sections (see Figure 5d–f) of each sample exhibited lamellar structures along the freezing direction. Lamellar pore structures are typical replicas of solidified aqueous suspensions with a low content of organic additives. Higher contents of PVA would influence the pore morphology [28]. The axial cross-sections showed the same trend as the other characterization methods with decreasing pore sizes with higher solid content. Figure 5d,e displays similar parallel pore channels. In Figure 5e, the crosslinking between the pore channels is increased and agrees with the mercury porosimetry results. The crosslinking structure develops due to the higher viscosity of the suspensions and hindering of the rearrangement of particles [29]. Figure 5f of the sample 20Y indicates a lamellar structure, but the pore channels are narrower, and the wall thickness increased.

**Figure 6:**
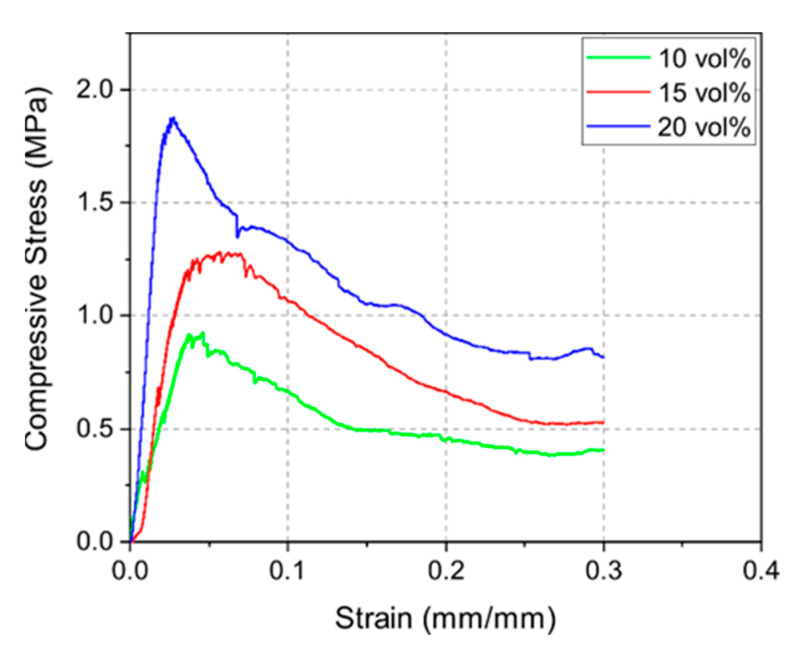
Exemplary stress–strain curves for sintered 10Y, 15Y, and 20Y samples for constant load rate of 1 N s^−1^.

Figure 6 displays the stress–strain curves of the 10Y, 15Y, and 20Y samples. The compressive strength measurements indicated a correlation between density and mechanical strength as well. Denser samples led to higher compressive strength. The highest mean compressive strengths for the 10Y, 15Y, and 20Y samples were 0.8, 1.3, and 1.6 MPa, respectively. The stress–strain curves also show the typical weakening of the strut structure of foams due to local buckling and strut failure [30]. After reaching a maximum stress level, when the integrity of the structure is maintained, the compressive stress decreases continuously due to sequential local failures of the structures [28,31]. Such curves are well displayed in this load-rate-driven experiment.

### 3.3. Effect of Thermal Aging

**Figure 7:**
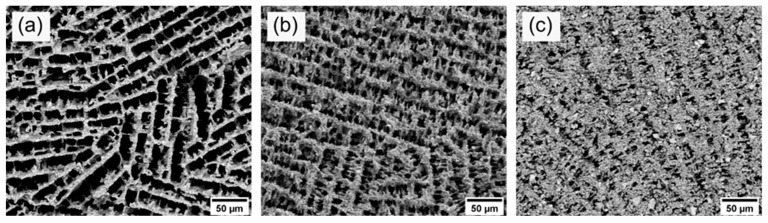
SEM images of the axial cross-section of samples 10Y (**a**), 15Y (**b**), and 20Y (**c**) after thermal aging at 1400 °C for 96 h.

To experimentally simulate the operating temperature during operation at ISOLDE, we carried out a second thermal treatment at 1400 °C for 96 h in air at standard pressure on the already sintered samples. The SEM images (see Figure 7) after thermal aging display the development of the pore morphology. The average pore sizes after thermal aging are also given in Table 3. In comparison to the pore size measurements before thermal aging, no significant change and degradation of the microstructure could be observed within the measurement uncertainties. Thus, the diffusion paths for the isotopes remained roughly the same during operation at 1400 °C for 96 h in air at standard pressure. Further studies will show whether the microstructure remains stable under typical ISOLDE conditions at high temperatures, low pressure and in an ISOLDE target container.

### 3.4. Thermo-Mechanical Calculations

**Figure 8:**
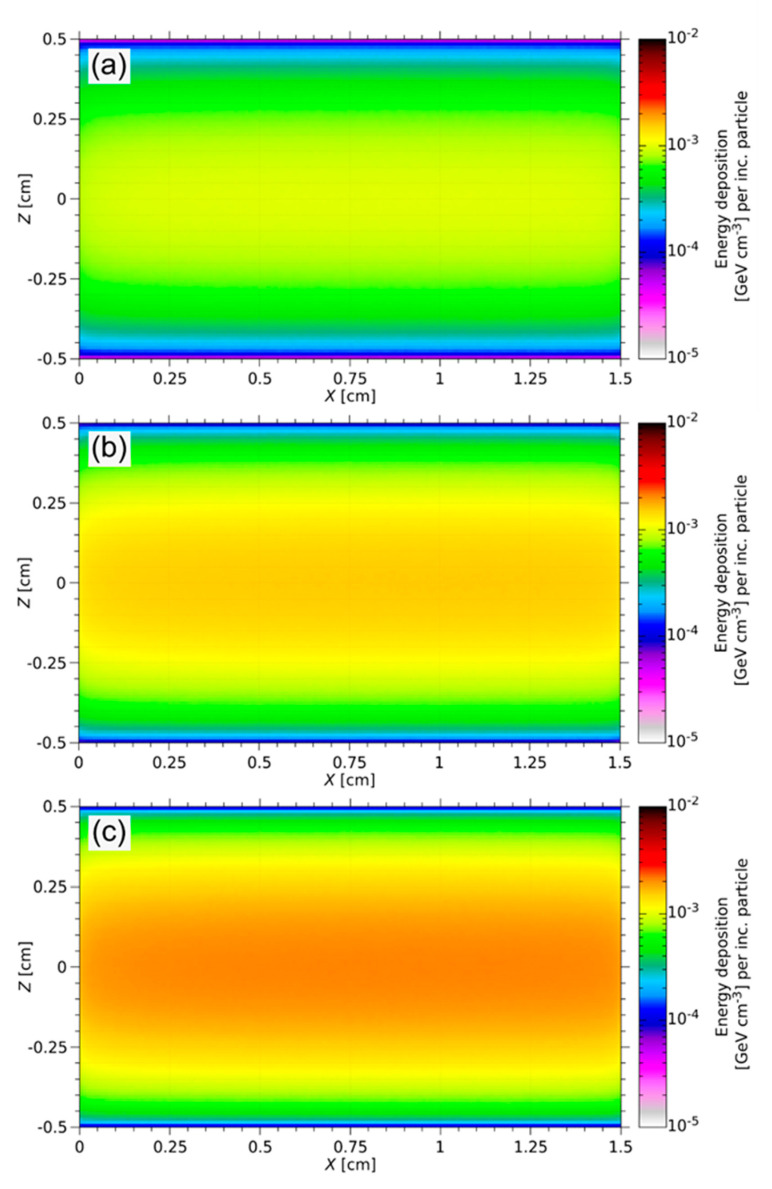
FLUKA simulation of the energy deposition in three different Y_2_O_3_ targets with 10 vol% (**a**), 15 vol% (**b**), and 20 vol% (**c**) solid loading for the ISOLDE beam. The FLUKA results are normalized per incoming particle (per inc. particle). The maximum energy deposited along the long axis of the cylinder is 1.68 × 10^−3^ GeV cm^−3^ per inc. particle (**a**), 2.58 × 10^−3^ GeV cm^−3^ per inc. particle (**b**), and 3.37 × 10^−3^ GeV cm^−3^ per inc. particle (**c**).

During the ISOL process, the targets degrade thermo-mechanically under high-energy irradiation [2]. The energy deposition of a 1.4 GeV proton beam at ISOLDE was simulated using FLUKA. Figure 8 shows the energy deposition spectrum of each sample. The proton beam hits the samples parallel to the long axis of the cylinder. Due to the Gaussian distribution of the proton beam width, the maximum energy deposition is in the center axis of the target. Along the beam axis, no significant decrease in beam intensity typically arises.

The maximum energy (*E_maxFluka_*) deposited per volume and incident particle permits estimation of the mechanical integrity of the targets. The energy deposited in the material per volume can be calculated as

(1) $$E_{dep}=E_{maxFluka}\;\cdot \;N_{ppp},$$

where *N_ppp_* is the number of protons per pulse of the ISOLDE beam, which is 3 × 10^13^ [8]. The deposited power (*P*) in the target volume (*V*) is determined based on varying repetition rates (*t*) and *E_dep_*,

(2) $$P=\frac{E_{dep}}{t}\cdot V.$$

The increase in temperature per pulse is computed as

(3) $$\Delta T=\frac{E_{dep}}{\rho \cdot c_{p}},$$

where *c_p_* is the specific heat capacity of yttrium oxide (544 J kg^−1^ K^−1^) [24], and ρ is the pure density of the prepared targets (see Figure 3). The mechanical integrity of the target material is evaluated by comparison of the thermal stresses and compressive strength of the porous scaffold structure. The calculation of the thermal stress involves Δ*T*, the measured Young modulus (*E_young_*) of the porous targets and the thermal expansion coefficient α of yttrium oxide (9.3 × 10^−6^ K^−1^ [32])

(4) $$\sigma =\Delta T\cdot E_{young\;}\cdot \alpha .$$

Table 4 summarizes the results of the thermo-mechanical calculations compared with the measured compressive strength (*σ*_s_) (see Figure 6). This comparison shows that the thermal stress per pulse has no effect on the mechanical integrity of the freeze-cast target material.

## 4. Conclusions

This study demonstrates that the fabrication of anisotropic yttrium oxide scaffolds by unidirectional solidification is feasible. Freeze casting of aqueous suspensions containing 10, 15, and 20 vol% solid loading shows different resulting porosities of the samples. The open porosity of the samples decreases with increased solid loading from 84 to 72%. The pore size measurements imply the same trend of decreasing pore size with higher solid loading. Mercury porosimetry as well as SEM images indicate that crosslinking between pore channels is more distinctive for samples with high solid loading. Thermal aging at 1400 °C for 96 h has shown no significant change in microstructure and pore size. Thermo-mechanical calculations, compared with compressive strength measurements, prove the mechanical stability of the scaffold structures during the ISOL application. Thus, this technique allows the production of tailored microstructures of yttrium oxide target materials with tunable pore structure and sizes as well as appropriate compressive strength and thermal stability for the ISOL process. Further studies will investigate the behavior and microstructural stability of the freeze-cast targets under typical ISOLDE conditions, i.e., at high temperatures, low pressure and inside an ISOLDE target container. In addition, equivalent studies on other materials, e.g., hafnia or thoria, are foreseen.

## Figures and Tables

**Table 1:** Composition of the suspensions with varying solid loading (content of additives in wt% with respect to ceramic powder).

Sample Labelling	10Y	15Y	20Y
Solid Loading (vol%)	10	15	20
PVA (wt%)	3	3	3
PEG (wt%)	2	2	2
Dispersant (wt%)	1.5	1.5	1.5

**Table 2:** Summary of the shrinkage of the samples after sintering at 1400 °C for 4 h.

Sample	Diameter (%)	Height (%)
10Y	6.5 ± 1.2	8.4 ± 0.7
15Y	6.5 ± 0.8	8.0 ± 0.5
20Y	5.9 ± 0.4	7.4 ± 1.3

**Table 3:** Summary of pore size measurements determined from SEM images before and after thermal aging at 1400 °C for 96 h.

Sample	Average Pore Size	
	Before Aging (µm)	After Aging (µm)
10Y	11.8 ± 3.6	11.1 ± 3.5
15Y	6.5 ± 2.0	7.1 ± 2.1
20Y	4.4 ± 1.4	5.2 ± 1.8

**Table 4:** Results of the thermo-mechanical analysis.

Sample	*E*_dep_ (J cm^−3^)	*P* (W)	Δ*T* (K)	*E*_young_ (MPa)	*σ* (MPa)	*σ*_s_ (MPa)
10Y	8.07	9.51	24.73	3.53	8.12 × 10^−3^	0.84
15Y	1.24	14.61	25.05	4.99	1.16 × 10^−2^	1.28
20Y	1.62	19.10	24.48	11.52	2.66 × 10^−2^	1.57

## Data Availability

The data presented in this study are available on request from the corresponding author.
